# *Bradyrhizobium elkanii nod* regulon: insights through genomic analysis

**DOI:** 10.1590/1678-4685-GMB-2016-0228

**Published:** 2017-07-31

**Authors:** Luciane M. P. Passaglia

**Affiliations:** Departamento de Genética, Instituto de Biociências, Universidade Federal do Rio Grande do Sul (UFRGS), Porto Alegre, RS, Brazil

**Keywords:** Bradyrhizobium, NodD_1_ protein, *nod* box, *nod* genes

## Abstract

A successful symbiotic relationship between soybean [*Glycine* *max* (L.) Merr.] and *Bradyrhizobium* species requires expression of the bacterial structural *nod* genes that encode for the synthesis of lipochitooligosaccharide nodulation signal molecules, known as Nod factors (NFs). *Bradyrhizobium diazoefficiens* USDA 110 possesses a wide nodulation gene repertoire that allows NF assembly and modification, with transcription of the *nodYABCSUIJnolMNOnodZ* operon depending upon specific activators, *i.e.*, products of regulatory *nod* genes that are responsive to signaling molecules such as flavonoid compounds exuded by host plant roots. Central to this regulatory circuit of *nod* gene expression are NodD proteins, members of the LysR-type regulator family. In this study, publicly available *Bradyrhizobium elkanii* sequenced genomes were compared with the closely related *B. diazoefficiens* USDA 110 reference genome to determine the similarities between those genomes, especially with regards to the *nod* operon and *nod* regulon. Bioinformatics analyses revealed a correlation between functional mechanisms and key elements that play an essential role in the regulation of *nod* gene expression. These analyses also revealed new genomic features that had not been clearly explored before, some of which were unique for some *B. elkanii* genomes.

## Introduction

Soybean symbiotic partners mainly belong to the genus *Bradyrhizobium*, initially proposed as a group of slow-growing, alkaline-producing root nodule nitrogen-fixing bacteria (Somasegaran and Hoben, 1994). Genetic and physiological information, including biochemical profile, DNA homology, and phylogenomics, is essential for clarifying the differences among isolates and for supporting the taxonomic classification of over 25 species in the genus *Bradyrhizobium* (Jordan, 1982; Rivas *et al.*, 2009). Among the species of this genus, considerable research efforts have focused on *B. japonicum* and *B. elkanii* because of their commercial use as a source for inoculant formulations, with many effective long-term programs for elite strain identification and selection being undertaken in different countries (Campos *et al.*, 2001; Kober *et al.*, 2004; Melchiorre *et al.*, 2011).

The characterization of symbiosis itself is not an easy task because of the high degree of specialization involved in this phenomenon, particularly since a restricted group of rhizobial species/strains interacts with only a select range of plant species/varieties and vice versa. Moreover, specificity may occur at distinct stages of the interaction, from the very initial bacterial contact with the plant root to late nodule development and nitrogen fixation, resulting in biological nitrogen fixation that varies according to the host-microsymbiont combination (Schumpp and Deakin, 2010; Wang *et al.*, 2012). In view of this, symbiosis can be conceived as a complex framework promoted by strong evolutionary forces that involves a stringent initial molecular dialogue and signal exchange between the symbiotic partners. The establishment of a successful mutualistic relationship initially requires products of the bacterial structural *nod* genes that encode for the synthesis of lipochitooligosaccharide (LCO) nodulation signaling molecules, also known as Nod factors (NFs). Subsequently, the recognition of symbiotic NFs by the plant triggers a signaling cascade that ultimately allows bacterial infection and induces *de novo* organogenesis of the nodule to accommodate the symbiont and further support nitrogen fixation (Tóth and Stacey, 2015). *Bradyrhizobium diazoefficiens* USDA 110 (formerly *B. japonicum* USDA 110) has a wide nodulation gene repertoire involved in NF assembly and modification.

In several species of rhizobia *nod* genes are frequently organized in an operon (*nod* operon), which suggests that regulation of their expression involves common mechanisms (Denarie *et al.*, 1993). Indeed, transcription of the *nodYABCSUIJnolMNO* operon in *B. diazoefficiens* USDA 110 depends upon transcriptional activators, *i.e.*, products of regulatory *nod* genes responsive to signaling molecules, such as flavonoid compounds, exuded by host plant roots (Luka *et al.*, 1993; Loh and Stacey, 2003). Central to this regulatory circuit of *nod* gene expression are NodD proteins, members of the LysR-type regulator family. Upon activation by a particular flavonoid ligand NodD proteins can bind to specific DNA motifs upstream of the *nod* operon, the so called *nod* boxes, and selectively control the expression of structural *nod* genes in the early stages of plant-bacteria interaction (Hong *et al.*, 1987; Henikoff *et al.*, 1988; Goethals *et al.*, 1992). Although multiple isoforms of NodD proteins have been identified in distinct rhizobial species, perhaps indicative of a role in expanding the plant host spectrum of these symbionts, only two were found in the *B. diazoefficiens* USDA 110 genome, namely, NodD_1_ and NodD_2_, products of the *nodD*_*1*_ and *nodD*_*2*_ genes, respectively (Gottfert *et al.*, 1989). These two proteins show distinct expression patterns and play different functional roles in regulating the expression of structural *nod* genes. Active NodD_1_, *i.e.*, in the presence of a flavonoid ligand molecule such as genistein in soybean root exudates, operates as a positive transcriptional regulator of the *nod* operon in *B. diazoefficiens*. Unique to this organism when compared to other known rhizobial species, NodD_1_ is not constitutively expressed; instead, it is induced by flavonoid compounds and shows autoregulation (Banfalvi *et al.*, 1988). Although nodulation requires *nod* gene induction by flavonoids in most diazotrophs, the efficiency of this process depends on appropriate spatial and temporal expression of these genes. Hence, it is not surprising that, in addition to positive transcriptional regulators, *nod* genes are also controlled by repressor elements, as is the case for NodD_2_ protein, that acts as a negative regulator of the *nod* operon (Loh and Stacey, 2003).

In *B. diazoefficiens* USDA 110, the core regulatory mechanism involving NodD_1_ and NodD_2_ is extended with additional regulators that act synergistically with NodD proteins to modulate the expression of *nod* genes. Of these, the roles of NolA, a MerR-type regulator encoded by *nolA*, and NodVW, the product of *nodVW,* that form a two-component regulatory system are particularly noteworthy. Initially identified as a soybean genotype-specific nodulation factor, NolA was later shown to be an activator of *nodD*_*2*_ involved in negative regulation with NodD_2_, with both involved in the feedback and quorum regulation of *nod* genes (Sadowsky *et al.*, 1991; Garcia *et al.*, 1996; Loh and Stacey, 2003). On the other hand, the NodVW two-component regulatory system provides an alternative flavonoid responsive pathway for *nod* gene activation, which explains the residual nodulation of soybean plants in NodD_1_ mutants (Göttfert *et al.*, 1990; Loh *et al.*, 1997).

As the major source for the soybean inoculant industry, strains of *B. japonicum* and *B. elkanii* differ markedly in their physiology and in their competitive fitness (Minamisawa, 1989; Vasilas and Furhmann, 1993). Such differences indicate the need to identify and characterize the genetic nature of specificity in the symbiotic relationship as a crucial step in developing guided strategies to enhance the effectiveness of soybean inoculants, given that biological nitrogen fixation ultimately provides a resource for more sustainable agricultural systems.

While there is a considerable amount of knowledge regarding the genetics and molecular mechanisms of the soybean symbiont *B. diazoefficiens*, including the complete genome sequence, information on the genetics of *B. elkanii* is restricted mainly to research focused on specific features. Although genomic data and a few drafts of the genome are available for *B. elkanii*, comparative genomic analyses of these two species have not been reported. In this study, publicly available *B. elkanii* genomes were compared with the closely related *B. diazoefficiens* USDA 110 reference genome (Kaneko *et al.*, 2002) to gain some insights into the mechanisms of *nod* gene expression in *B. elkanii*, especially for the *nod* operon and *nod* regulon. The results of this analysis should provide a more comprehensive understanding of the molecular dynamics and complexity of mechanisms involved in fine-tuning signal communication between this symbiont and its host plants.

## Material and Methods

### Strains and genomic data

Genomic data consisting of the complete genome of *B. diazoefficiens* USDA 110 (reference genome) and draft genomes of *B. elkanii* strains SEMIA 587, CCBAU 05737, CCBAU 43297, USDA 94, USDA 3254 and USDA 3259 were obtained from the publicly available database of The National Center for Biotechnology Information (NCBI; http://www.ncbi.nlm.nih.gov). Genome features and accession numbers are described in Table 1. The type-strain taxonomy of each organisms genome was confirmed by 16S rRNA sequence analysis using the RDP SeqMatch k-nearest-neighbor (k-NN) classifier (Wang *et al.*, 2007) and checked with Basic Local Alignment Search Tool (BLAST) searches and pairwise global sequence alignments implemented in the EzTaxon server database (Kim *et al.*, 2012).

**Table 1:** Characteristics of the genomes analysed.

	*B. diazoefficiens*	*B. elkanii*	*B. elkanii*	*B. elkanii*	*B. elkanii*	*B. elkanii*	*B. elkanii*
	USDA 110	SEMIA 587	CCBAU 05737	CCBAU 43297	USDA 94	USDA 3254	USDA 3259
Geographic location	USA	Brazil	China	China	USA	USA	USA
Number	1*	2,431	751	654	174	87	102
of contigs							
Size (Mb)	9.11	8.68	9.77	9.35	9.56	8.98	8.72
Av. read coverage	-	32x	109x	120x	NA**	NA	NA
N50 (kb)	-	6,929	25,010	25,032	138,573	249,311	245,986
G+C (%)	64.1	63.6	63.5	63.8	63.7	63.8	63.9
Number of	3 rRNA	3 rRNA	3 rRNA	3 rRNA	3 rRNA	3 rRNA	3 rRNA
RNA calls	49 tRNA	40 tRNA	47 tRNA	48 tRNA	47 tRNA	47 tRNA	47 tRNA
Number of CDS calls	8648	8236	9396	8928	9122	8565	8310
NCBI BioSample	GCF000011365.1	SAMN02471378	SAMN02469485	SAMN02469464	SAMN02584917	SAMN02441448	SAMN02440775
NCBI BioProject	PRJNA17	PRJNA86995	PRJNA77219	PRJNA77219	PRJNA165317	PRJNA165319	PRJNA162999
NCBI assembly accession	GCA_000011365.1	GCA_000257685.1	GCA_000261505.1	GCA_000261525.1	GCA_000519225.1	GCA_000472765.1	GCA_000473005.1

### Bioinformatics analyses

Functional annotation was done with the Rapid Annotation using Subsystem Technology (RAST) server (Aziz *et al.*, 2008), with Glimmer set for gene calling (Salzberg *et al.*, 1998), frameshift correction, backfilling of gaps and automatic fixing errors. Assigned functional features were triple-checked with InterProScan (Zdobnov and Apweiler, 2001) by the signature-recognition method in the InterPro database (Hunter *et al.*, 2009), ScanProsite (de Castro *et al.*, 2006) for protein signature matches in the PROSITE database (Sigrist *et al.*, 2010), and BLASTp against the UniProtKB database (Magrane and Consortium, 2011). An inventory of genes involved in nodulation (structural- and regulatory-*nod*/*nol* genes) for each genome in this study is provided in Table S1. The *B. diazoefficiens* USDA 110 genome (Kaneko *et al.*, 2002) was used as the reference genome and missing *nod*/*nol* genes were searched for in other genomes with BLASTn using homologous nucleotide sequences of the closely-related reference species. Possible frameshift annotation errors in assigned genes were corrected and Open Reading Frames (ORFs) were checked with the Expert Protein Analysis System (ExPASy) translate tool (Gasteiger *et al.*, 2003) by comparing with the respective reference.

The subcellular localization of proteins was predicted using sequence-based tools in a coordinated fashion (Emanuelsson *et al.*, 2007). Initially, SignalP 4.1 software (Petersen *et al.*, 2011) was used to screen amino acid sequences for the presence and location of signal peptide cleavage sites characteristic of secretory proteins and this was followed by the *ab initio* prediction of non-classical protein secretion, *i.e.*, secretion not triggered by a signal peptide, with SecretomeP 2.0 (Bendtsen *et al.*, 2004). Lipoprotein signal peptides and N-terminal membrane helix prediction was done using LipoP 1.0 (Rahman *et al.*, 2008), while the presence and location of potential twin-arginine translocation signal peptide cleavage sites was verified with TatP 1.0 (Bendtsen *et al.*, 2005). Finally, the prediction of transmembrane topology in proteins was assessed using a combined approach based on a hidden Markov model algorithm implemented in the TMHMM 2.0 server (Krogh *et al.*, 2001) and Phobius (Käll *et al.*, 2004).

All bioinformatics analyses used the default parameters of the respective software.

### Phylogenetic and sequence analyses

Multiple sequence alignment of NodD proteins was done by distance estimation using *k*mer counting and progressive alignment with log-expectation scores, followed by refinement using tree-dependent restricted partitioning implemented by MUSCLE (Edgar, 2004). The algorithm was implemented in the Molecular Evolutionary Genetics Analysis - MEGA 6.0 package (Tamura *et al.*, 2013) and the parameters were set for the Neighbor-Joining clustering method in all interactions, with −2.9 and 0 for gap opening and gap extension penalties, respectively (center specified as 0). Subsequent phylogenetic analysis and tree reconstruction were done using the Neighbor-Joining method in the same package, with the molecular distances of the aligned sequences computed based on p-distance parameters and 1,000 bootstrap replicates and pairwise deletion treatment for gaps. Point accepted mutation (PAM) 250 calculations were used as a substitution matrix model for scoring sequence alignments (Dayhoff and Schwartz, 1978).

### Protein structure prediction and alignment

Protein 3D structure prediction was done using the SWISS-MODEL web server (Biasini *et al.*, 2014) based on evolutionarily-related structures, amino acid sequences and protein structure homologies. The technique uses hidden Markov model-sensitive searches run against the SWISS-MODEL template library (SMTL) to generate a structural model of the protein of interest. A QMEAN potential to assess model quality is then generated with an independent accuracy evaluation by the Continuous Automated Model EvaluatiOn project – CAMEO (Haas *et al.*, 2013) based on target sequences pre-released by the Protein Data Bank (PDB) (Berman *et al.*, 2007). The predicted protein structures were aligned with a combinatorial extension (CE) algorithm (Shindyalov and Bourne, 1998) implemented in the Research Collaboratory for Structural Bioinformatics (RCSB) PDB Protein Comparison Tool at www.rcsb.org (Bernstein *et al.*, 1977; Berman *et al.*, 2007; Goodsell *et al.*, 2015). The parameters were set as 30 for the maximum gap size allowed during aligned fragment pairs (AFP) extension in fragment size m = 8, with gap open and gap extension penalties of 5.0 and 0.5, respectively.

## Results

### *nod* operon and regulatory genes

**Figure 1:**
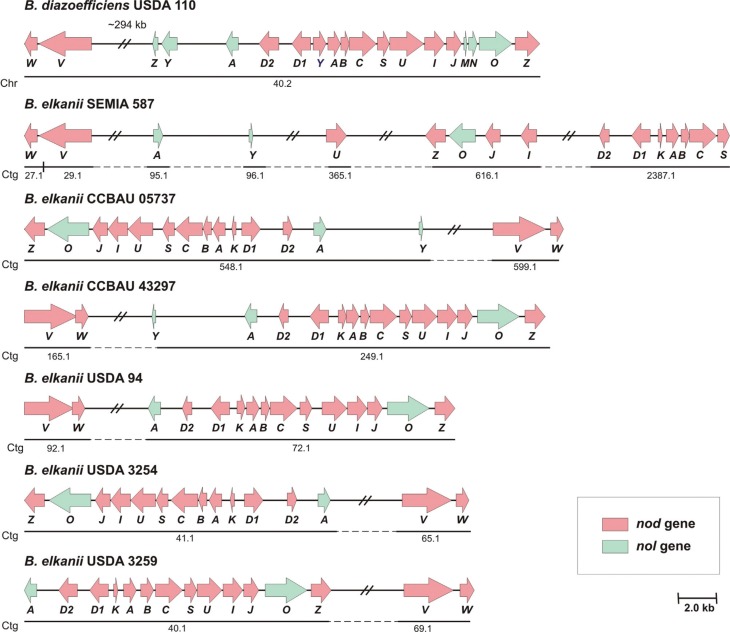
Genetic map showing organization of the *nod*/*nol* genes in *B. diazoefficiens* USDA 110 (reference genome) and six *B. elkanii* genomes. Letters indicate the name of each gene in the genome. Gene classes are color coded and transcriptional orientation is indicated by the arrows. Relative gene distances are indicated or follow the scale presented. Gene location within the genome is represented by a straight line below the gene map of the respective chromosome (*B. diazoefficiens*) or contig (*B. elkanii*) ID.

Annotation of the *B. elkanii* genome followed by manual curation revealed a conserved operon structure and organization of the *nodKABCSUIJnolOnodZ* genes in strains CCBAU 05737, CCBAU 43297, USDA 94, USDA 3254 and USDA 3259 similar to that in the *B.* *diazoefficiens* USDA 110 *nod* operon, with the substitution of *nodY* for the corresponding *nodK* and the lack of *nolMN* genes. Curiously, in *B. elkanii* SEMIA 587, a distinct pattern for this “canonical” gene organization was observed, with these genes scattered in the chromosome and organized as *nodKABCS*, a second sparse block containing *nodI*, *nodJ*, *nolO* and *nodZ*, and then *nodU*, which was separate from the other genes of this operon. As in the case of *nolMN*, *nolZ* was not identified in the genomes of any *B. elkanii* strain; likewise, *nolY* was not detected in the genomes of strains USDA 94, USDA 3254, and USDA 3259 (Figure 1).

Annotation also highlighted the presence of *nodD*_*1*_ and *nodD*_*2*_ regulatory genes in all six *B. elkanii* genomes and in the *B. diazoefficiens* USDA 110 genome. The organization of these two genes in the *B. elkanii* genomes followed the pattern observed for *B. diazoefficiens* USDA 110, *i.e.*, they were positioned close to each other and close to the *nod* operon, although in opposite orientation. Similarly, the presence of *nodVW* that coded for the two-component regulatory elements was ubiquitous in all six *B. elkanii* genomes, with its location relative to the *nod* operon varying according to each genome. Conversely, the *nolA* gene showed a conserved location among the *B. elkanii* genomes, close to *nodD*_*2*_; the exception was for strain SEMIA 587, in which this gene was located at a position distant from *nodD*_*2*_ (Figure 1).

### Analysis of *nodD* promoter regions

**Figure 2:**
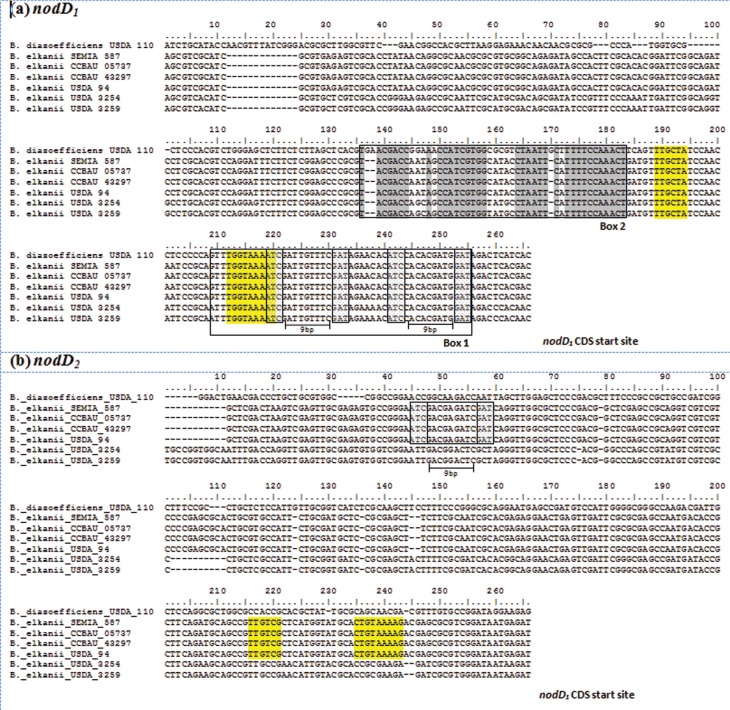
Alignment of the ~250-bp region upstream from *nodD* ORFs in *Bradyrhizobium* genomes. (a) The *nodD*_*1*_ *nod* box sequences are boxed and show a consensus *nod* box sequence near the *nodD*_*1*_ ORF start codon (Box 1) and a presumptive *nod* box-like sequence (Box 2) upstream, with conserved residues highlighted in grey, and (b) *nodD*_*2*_ partial *nod* box sequence structure (boxed) found in some *B. elkanii* genomes. Smaller boxes denote the putative *nod* box motifs (ATC-N_9_-GAT) with their respective palindromic structure. Yellow shaded sequences show a −10/-35 σ^70^ potential promoter. Sequences read 5' to 3' from right to left, with the first nucleotide in the sequence representing that immediately prior to ATG from the *nodD*_*1*_ (a) or *nodD*_*2*_ (b) CDS start site

Analysis of a 250-bp region upstream of the *nodD*_*1*_ ORFs revealed that all six *B. elkanii* genomes and the *B. diazoefficiens* USDA 110 genome contained one −10/-35 σ^70^ potential promoter (TTGCTA-N_17_-TGGTAAAAT) located 46 bp upstream from the *nodD*_*1*_ CDS start site. Additionally, all sequences showed two 47-bp *nod* boxes, one of which corresponded to a consensus *nod* box sequence with the respective palindromic structure, located 11 bp from the *nodD*_*1*_ CDS start site that controls transcription of the *nod* operon. The second one was a presumptive *nod* box-like sequence located in the upstream region (84 bp) of the *nodD*_*1*_ CDS start site (Figure 2A). Interestingly, the consensus *nod* box sequence (Box1) overlapped the sequence of the −10/-35 σ^70^ putative *nodD*_*1*_ promoter, while the presumptive *nod* box-like sequence was located just 6 bp upstream to it (Figure 2A).

On the other hand, analysis of the 250-bp region upstream of *nodD*_*2*_ revealed that only the *B. elkanii* SEMIA 587, CCBAU 05737, CCBAU 43297 and USDA 94 genomes exhibited a −10/-35 σ^70^ potential promoter with the sequence TTGTCG-N_13_-CTGTAAAAG, along with a partial *nod* box-like sequence located 152 bp upstream from the putative σ^70^ promoter. In the *B. diazoefficiens* USDA 110 and *B. elkanii* USDA 3254 and USDA 3259 genomes, neither of these elements could be identified in the *nodD*_*2*_ promoter region, *i.e.*, they apparently had no potential −10/-35 σ^70^ promoters nor a partial (or complete) *nod* box-like sequence (Figure 2B).

The *nolA* promoter regions (250 bp) of all genomes in this study exhibited a −10/-35 σ^70^ potential promoter sequence (TTGAAT-N_16_-TTGTAGGCT), except for *B. elkanii* SEMIA 587. Additionally, apart from *B. elkanii* SEMIA 587, the *nolA* promoter regions displayed a high degree of sequence similarity among genomes (Figure S1A). Potential −10/-35 σ^70^ promoter sequences for the *nodVW* regulatory gene were found in all genomes, although there were differences in the structure and location of these promoters. In contrast to *nolA*, the promoter regions of the *nodVW* genes differed considerably in sequence conservation among genomes (Figure S1B). A search for the *nod* box in the *nolA* and *nodVW* promoter regions revealed the absence of this regulatory motif in the 250-bp upstream gene sequences in all genomes (Figure S1A,B).

### Sequence analysis of regulatory Nod proteins

All the *B. elkanii* genomes displayed NodD_1_ ORFs of 314 amino acids, in agreement with the size of the NodD_1_ protein of *B. diazoefficiens* USDA 110. In contrast, there was discrete variation in the size of the NodD_2_ ORFs among genomes, with ORFs of 330 amino acids in *B. diazoefficiens* USDA 110, 331 amino acids in *B. elkanii* strains SEMIA 587, CCBAU 05737, CCBAU 43297 and USDA 94, and 329 residues in *B. elkanii* strains USDA 3254 and USDA 3259 (Table S1).

NodD_1_ and NodD_2_ showed a relatively high degree of conservation, although some dissimilarity among organisms ultimately clustered each *B. elkanii* protein into one of two groups (Figure S2). Indeed, a PAM250 matrix showed that *B. elkanii* strains SEMIA 587, CCBAU 05737, CCBAU 43297 and USDA 94 shared 100% global sequence similarity among themselves for both NodD_1_ and NodD_2_; consequently, each protein set for these strains grouped together. The same situation occurred with USDA 3254 and USDA 3259, although sequence similarity decreased by 7% for NodD_1_ and by 12% for NodD_2_ compared to their respective homologs from other strains within the same species. NodD regulator proteins from the *B. elkanii* strains were still conserved when compared to *B. diazoefficiens* USDA 110 orthologs, especially NodD_1_, which exhibited at least 93% sequence similarity, while for NodD_2_ no less than 73% similarity was observed (Figure S3A-B). Additionally, conservation plots for NodD_1_ and NodD_2_ proteins from the *B. elkanii* genomes revealed lower conservation at the carboxy-terminus region of the proteins, especially for NodD_2_, when compared to the reference genome of *B. diazoefficiens* USDA 110 (Figure S4A,B).

**Figure 3:**
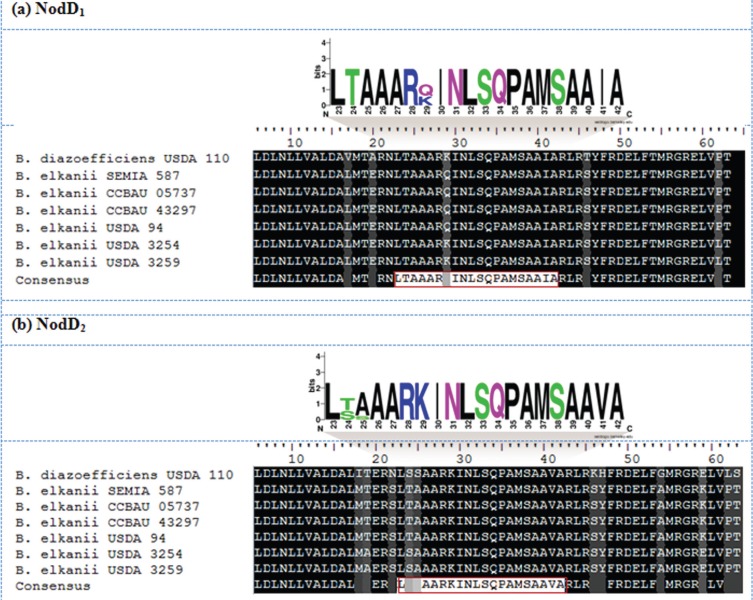
Multiple sequence alignment (MSA) of the *B. elkanii* NodD HTH LysR-type domain and sequence logo showing conservation of the HTH DNA-binding motif. The HTH DNA-binding sequence conserved among organisms is boxed in red. The sequences are for the (a) NodD_1_ and (b) NodD_2_ domains.

All the sequences analysed belonged to the LysR family of transcriptional regulators, and exhibited a 57-amino acid helix-turn-helix (HTH) LysR-type domain located at position 6-63. These domains also contained a 20-amino acid HTH DNA-binding motif from residues 23-42 in the amino-terminus region of NodD_1_ and NodD_2_ proteins. The NodD_1_ DNA-binding motif from *B. elkanii* strains SEMIA 587, CCBAU 05737, CCBAU 43297 and USDA 94 was highly conserved and showed 100% identity with the sequence LTAAARQINLSQPAMSAAIA (Figure 3A). Curiously, *B. elkanii* USDA 3254 and USDA 3259 showed 100% identity with the *B. diazoefficiens* USDA 110 NodD_1_ DNA-binding motif LTAAARKINLSQPAMSAAIA, with a single amino acid substitution occurring at position 29, in which glutamine (Q) was replaced by lysine (K) (Figure 3A).

The *B. elkanii* NodD_2_ DNA-binding motif also split into two groups, with strains SEMIA 587, CCBAU 05737, CCBAU 43297 and USDA 94 showing 100% identity for the sequence LTAAARKINLSQPAMSAAVA, while for strains USDA 3254 and USDA 3259 a serine (S) replaced the threonine (T) at position 24 of NodD_2_, resulting in the DNA-binding motif LSAAARKINLSQPAMSAAVA (Figure 3B). A comparison of NodD_2_ among *Bradyrhizobium* species showed that *B. diazoefficiens* USDA 110 shared higher similarity with *B. elkanii* strains USDA 3254 and USDA 3259, although an additional amino acid residue divergence at position 25 was observed, with replacement of an alanine (A) in *B. elkanii* for a serine (S) in *B. diazoefficiens*, providing the sequence LSSAARKINLSQPAMSAAVA (Figure 3B).

*In silico* analysis of the subcellular location of NodD_1_ and NodD_2_ proteins did not predict the presence of any significant ordinary signal peptide cleavage site in their amino acid sequences or any twin-arginine signal peptide cleavage site. Lipoprotein signal peptides and non-classical protein secretory patterns were also not identified. Additional protein topology and signal peptide examination did not identify any transmembrane helices. Finally, calculations using an integrative approach algorithm indicated that NodD_1_ and NodD_2_ were more likely to be cytoplasmic components rather than membrane-bound proteins.

### Structural analysis of regulatory Nod proteins

Global protein structure alignment of NodD_1_ from the *B. elkanii* strains revealed high identity and similarity to NodD_1_ protein from the reference genome of *B.* *diazoefficiens* USDA 110. In particular, the genomes of *B. elkanii* strains USDA 3254 and USDA 3259 showed 92.62% and 97.65% structural identity and similarity for NodD_1_ compared to the orthologous protein from *B.* *diazoefficiens* USDA 110, respectively. Although slightly lower, the corresponding values for NodD_1_ structural identity and similarity from *B. elkanii* strains SEMIA 587, CCBAU 05737, CCBAU 43297 and USDA 94 were 91.61% and 95.97%, respectively. Analysis of the NodD_1_ structural alignments indicated that the higher identity and similarity of *B. elkanii* strains USDA 3254 and USDA 3259 with *B.* *diazoefficiens* USDA 110 resided mainly in the HTH motif of the DNA-binding domain (Figure S5A-F). In contrast, NodD_2_ global protein structure alignments for the *B. elkanii* SEMIA 587, CCBAU 05737, CCBAU 43297 and USDA 94 genomes showed higher identity (75.17%) and similarity (84.56%) with the NodD_2_ ortholog from *B.* *diazoefficiens* USDA 110 than with the *B. elkanii* USDA 3254 and USDA 3259 genomes, for which the identity and similarity were no higher than 74.16% and 82.89%, respectively (Figure S6A-F). Additionally, even though substantial identity and similarity were observed in the structural alignment of NodD_2_ between *Bradyrhizobium* species, the values were lower compared to the structural alignments for NodD_1_ proteins.

**Figure 4:**
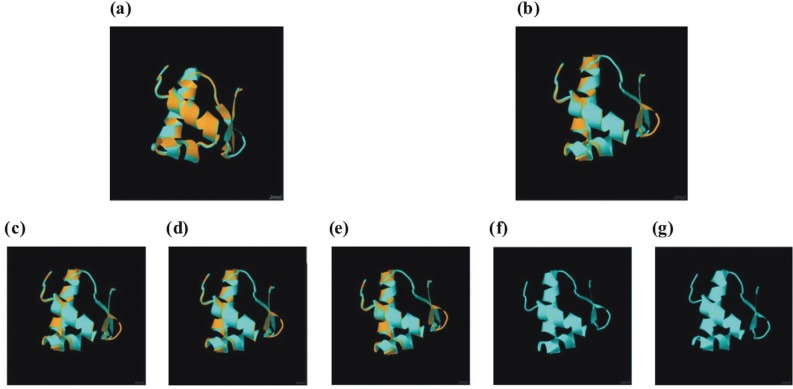
Structural alignment of the NodD_1_ and NodD_2_ 57-residue HTH domain within each genome using the Combinatorial Extension (CE) algorithm. The alignments are for *B. diazoefficiens* USDA 110 (a), *B. elkanii* SEMIA 587 (b), *B. elkanii* CCBAU 05737 (c), *B. elkanii* CCBAU 43297 (d), *B. elkanii* USDA 94 (e), *B. elkanii* USDA 3254 (f) and *B. elkanii* USDA 3259 (g). Orange residues represent NodD_1_ superimposed on light-blue residues representing NodD_2_.

When the global structural alignment of NodD_1_ *vs.* NodD_2_ proteins was compared within each genome, the identity and similarity were still relatively significant. In the *B. elkanii* USDA 3254 and USDA 3259 genomes for example, the NodD_1_ *vs.* NodD_2_ structural alignment showed 70.47% identity and 81.88% similarity, while for the *B. elkanii* SEMIA 587, CCBAU 05737, CCBAU 43297 and USDA 94 genomes, the identity and similarity reached values of 69.46% and 81.54%, respectively. The *B. diazoefficiens* USDA 110 reference genome showed the most dissimilar structural alignment of NodD_1_ *vs.* NodD_2_ and consequently had the lowest identity (63.76%) and similarity (80.54%) values (Figure S7A-F). However, considering only the 57-residue protein segment corresponding to the DNA-binding domain containing the HTH motif, the NodD_1_ *vs.* NodD_2_ structural alignment values were higher in all genomes, being identical in the *B. elkanii* USDA 3254 and USDA 3259 genomes, *i.e.*, 100% identity and similarity. The *B. elkanii* SEMIA 587, CCBAU 05737, CCBAU 43297 and USDA 94 genomes also showed better structural alignment of the amino-terminus region that accommodated the DNA-binding domain for the NodD_1_ *vs.* NodD_2_ comparison, with 91.38% identity and 98.28% similarity, compared to *B. diazoefficiens* USDA 110, which showed values of 81.03% and 93.10% for identity and similarity, respectively (Figure 4).

## Discussion

### *nod* operon/regulon in *Bradyrhizobium* genomes

Lipochitooligosaccharides (LCOs) produced by the action of bacterial nodulation (*nod*, *nol*, *noe*) gene products are key signaling molecules in establishing the *Bradyrhizobium*-legume symbiosis that triggers the formation of a new organ (the nodule) in which biological nitrogen fixation occurs (Day *et al.*, 2000). *Bradyrhizobium* *diazoefficiens* and *B. elkanii* can nodulate soybean plants. Despite this common ability, detailed genetic and biochemical studies involving different *Bradyrhizobium* species have shown distinct physiological profiles that ultimately affect their symbiotic behavior, including nodulation capacity, nitrogen fixation efficiency and competitive ability for nodule formation (Kober *et al.*, 2004). Most of the structural and regulatory *nod*/*nol* genes with known function seem to be conserved between the *B. diazoefficiens* USDA 110 and *B. elkanii* genomes. Moreover, the presence of the structural *nodY/KABCSUIJnolOnodZ* genes, arranged in an operon in the *B. diazoefficiens* and most *B. elkanii* genomes, may indicate that similar patterns of LCO formation can be expected for these organisms, depending primarily upon a particular operon regulation in each genome.

The organization of *nod* genes in clusters/operons is a common and often conserved feature among various rhizobia genomes, many of them with a chromosomal location, as in the case of *B. diazoefficiens*, thereby facilitating coordination of the expression of these respective genes (Schlaman *et al.*, 1998). Although *nod* genes in *Bradyrhizobium* are frequently organized in operons, this is not a fixed pattern, as seen in the *B. elkanii* SEMIA 587 genome which showed a reduced *nodKABCS* operon with additional *nod*/*nol* genes scattered along the chromosome. Interestingly, separation of the genes from this operon may result in the differential regulation of *nod* genes, ultimately providing an altered pattern of LCO formation for this strain compared to other *B. elkanii* genomes, even though this is not mandatory for divergent *nod* gene arrangement (Vázquez *et al.*, 1991). As in most rhizobia, expression of the *nod*/*nol* operon in *Bradyrhizobium* is dependent on distinct flavonoid compounds produced and secreted by each leguminous host plant, with the quantity and spectrum of these molecules varying according to the age and physiological state of the plant (Phillips and Streit, 1996; Loh and Stacey, 2003; Hassan and Mathesius, 2012).

Regulation of the *nod/nol* operon in *B. diazoefficiens* and *B. elkanii* is essentially under control of the same elements, namely, the *nod* regulon that consists of *nodD*_*1*_, *nodD*_*2*_, *nolA* and *nodVW* genes, all of them present in the genomes of both species. The pattern of gene organization observed for *nodD*_*1*_ and *nodD*_*2*_, both of which were invariably located close to each other and in opposite orientation to the *nod* operon in *Bradyrhizobium* genomes, reinforced their importance as key components in the core regulatory mechanism of operonic *nod* gene expression. Indeed, the products of *nodD* genes are assumed to be fundamental in coordinating structural *nod* genes in many symbionts, including *Bradyrhizobium* species (Mulligan and Long, 1989; Honma *et al.*, 1990; Loh and Stacey, 2003; del Cerro *et al.*, 2015). For instance, in *B. diazoefficiens* products of the *nodD*_*1*_ and *nodD*_*2*_ regulatory genes orchestrate *nodYABCSUIJnolMNOnodZ* operon expression by activation or repression, respectively (Loh and Stacey, 2003). Such a regulatory system suggests a common mechanism that may apply for *B. elkanii* genomes with conserved *nod* gene organization.

Besides their divergent transcriptional orientation, components of the LysR-type transcriptional regulator (LTTR) family, such as NodD_1_ and NodD_2_, also exhibit another characteristic feature, namely, autoregulation (Maddocks and Oyston, 2008), which ultimately indicates that expression of the associated operon largely depends on the regulation of these regulatory genes themselves. Since *B. diazoefficiens* *nodD* mutants retain a large marginal ability to nodulate soybean plants, alternative transcriptional activators were proposed to take part in this process. Indeed, the identification of a two-component regulatory system consisting of NodV and NodW, products of *nodVW,* sheds some light on this paradox and provides a suitable explanation. In such a system, the sensor kinase component NodV can detect the environmental stimulus (such as a specific isoflavone) resulting in its autophosphorylation and subsequent signal transduction by transfer of the phosphoryl group to its cognate response regulator protein NodW, which in turn is then able to activate its target *nod* operon (Loh *et al.*, 1997). Further evidence that *Bradyrhizobium* *nodVW* products are essential for efficient nodulation of mung bean [*Vigna radiata* (L.) Wilezek], cowpea [*Vigna unguiculata* (L.) Walp.] and siratro [*Macroptilium atropurpureum* (Moc. & Sessé ex DC.) Urb.], but are not required for soybean, suggests a host-specific role for these proteins, probably through recognition of specific flavonoid inducers produced by the host plants in response to the correlate sensor component NodV (Göttfert *et al.*, 1990; Sanjuan *et al.*, 1994). As shown here, we identified this same system in *B. elkanii* genomes, suggesting that both species possess an alternative pathway for *nod* operon activation and possibly a strategy for broadening their respective host ranges.

Whereas successful soybean nodulation depends on timely expression of the structural *nod* gene at the right place and in suitable amounts, it is reasonable to assume that negative regulation also occurs, in addition to activation. Although this negative control in *B. diazoefficiens* is mediated by the *nodD*_*2*_ product that operates as a *nod* operon repressor as mentioned before, this process is assisted by the *nolA* gene product, which establishes an additional level of regulation by inducing *nodD*_*2*_ expression under appropriate conditions. In other words, the product of *nolA* acts as an transcriptional inducer of the repressor *nodD*_*2*_ (Garcia *et al.*, 1996). As already mentioned, NolA is a member of the MerR family of transcriptional regulators that activates transcription upon binding to specific DNA motifs and consequently induces DNA binding leading to its appropriate alignment for RNA polymerase positioning and subsequent transcriptional activation (Ansari *et al.*, 1992; Philips *et al.*, 2015). Moreover, *nolA* has the peculiar feature of encoding for the three proteins NolA_1_, NolA_2_ and NolA_3_ that originate from three in-frame ATG start codons, with NolA_1_ controlling the expression of the other two and being involved in the activation of *nodD*_*2*_ (Loh *et al.*, 1999). This mechanism is also shared with high homology by the *B. elkanii* genomes and, in most cases, shows a very similar gene organization and transcription to *nodD*_*1*_ and *nodD*_*2*_, as previously demonstrated (Dobert *et al.*, 1994). This finding indicates tight *nod* gene regulation in this species as well.

### *cis-regulatory* *nod* elements and *trans*-acting factors

Considering the nature of genomic elements present in the *nod* operon and especially in the *nod* regulon of *B. diazoefficiens* and their marked similarity with those in the *B. elkanii* genomes, both in terms of structure and organization, we hypothesized that major phenotypic differences in LCO production and secretory patterns in these two species could occur at some transcriptional or post-transcriptional regulatory level. Accordingly, in the presence of the corresponding flavonoid compounds, activated NodD proteins would specifically bind to conserved *cis*-regulatory elements on bacterial DNA, namely, the *nod* boxes. These regulatory structures basically consist of a 47-bp conserved region containing the *nod* box consensus sequence with the palindromic ATC-N_9_-GAT motif and are located upstream to the *nod* operon and control its expression (Rostas *et al.*, 1986; Nieuwkoop *et al.*, 1987; Goethals *et al.*, 1992). The conservation of such *cis*-regulatory elements and their essentiality in many rhizobia species studied so far strongly suggests that NodD_1_ is central in *nod* operon gene expression in these species, including *Bradyrhizobium* (Spaink *et al.*, 1987). The *nod* operon activation by NodD_1_ in *B. elkanii* genomes is no exception to this rule and seems to be under a similar, if not identical, regulatory mechanism since the same genetic features, highly conserved in relation to *B. diazoefficiens* USDA 110, are observed in this context.

Besides the activation of *nod* gene expression, an autoregulatory function unique to *Bradyrhizobium* is assigned to *nodD*_*1*_ by its own product NodD_1_ upon binding the same isoflavone molecules that activate the *nod* operon. Due to divergent transcriptional orientation, the *nod* box sequences are located upstream from the *nod* operon containing the structural *nod* genes and the *nodD*_*1*_/*nodD*_*2*_ regulatory genes. This location of *nod* boxes in between the *nod* operon and *nodD*_*1*_/*nodD*_*2*_ within the genome complicates the analysis of their regulation. Although autoregulation by NodD_1_ is also accomplished by a DNA-binding mechanism, this is reportedly achieved by binding to an alternative presumptive *nod* box-like sequence located upstream to the consensus *nod* box in *B. diazoefficiens* USDA 110 (Banfalvi *et al.*, 1988; Wang and Stacey, 1991). Indeed, the conservation of this regulatory sequence in *B. elkanii* genomes reinforces its proposed function, a conclusion supported by the close proximity of this additional *cis*-regulatory region to a −10/-35 σ^70^ potential promoter. Despite similarities such as high sequence conservation and location in the genome, a few differences still exist among the presumed *nod* box-like sequences in distinct *Bradyrhizobium* species, *e.g.*, the slightly smaller size and some nucleotide divergence in *B. elkanii* genomes compared to *B. diazoefficiens*, that ultimately may affect the DNA-binding process itself.

Based on the *nodD*_*1*_ and *nodD*_*2*_ sequence-homology and their resulting description as members of LysR-type regulators, it seems reasonable to also consider a potential autoregulatory role for NodD_2_, akin to what can be observed for NodD_1_. Previous screening of *Rhizobium japonicum* USDA 191 DNA sequences for the presence of a *nod* box revealed that only the *nodD*_*1*_ promoter region showed high sequence homology to other *nod* boxes, while no extensive similar motifs were identified upstream to *nodD*_*2*_, even though several alignments of up to four homologous base pairs were observed (Appelbaum *et al.*, 1988). Although no complete *nod* box has been found in the promoter region of *nodD*_*2*_ in *Bradyrhizobium* genomes, a remarkably conserved sequence containing only one copy of the palindrome ATC-N_9_-GAT, characteristic of a *nod* box sequence structure, was identified in some *B. elkanii* genomes. Curiously, the same genomes that contained this palindromic sequence also displayed a potential −10/-35 σ^70^ binding site. Since this relationship has not been reported before, the precise biological significance of this findings is still unclear and may represent another mechanism (in addition to NolA) by which *nodD*_*2*_ transcription is regulated in this species; this question deserves further investigation.

The current model for *nodD*_*2*_ expression in *B. diazoefficiens* essentially considers the induction of transcription by NolA upon binding to a putative NolA binding-site present upstream to *nodD*_*2*_ (Garcia *et al.*, 1996; Loh and Stacey, 2003). Sequence homology has shown that NolA is a member of the MerR transcriptional regulator protein family that is known to activate suboptimal σ^70^-dependent promoters through protein-dependent DNA distortion that ultimately provides an appropriate alignment of −10/-35 σ^70^ and the correct positioning of RNA polymerase in relation to the respective promoter (Brown *et al.*, 2003). Surprisingly, as shown here, preliminary screening for a potential NolA binding site upstream to *nodD*_*2*_ found no corresponding conserved *cis*-element in this region, contrary to current literature.

### NodD protein sequences and structure conservation

In addition to *cis*-regulatory regions in the bacterial genome, *trans*-acting factors can also affect transcriptional regulation. The high degree of protein conservation observed for NodD_1_ and NodD_2_ among *Bradyrhizobium* genomes highlighted the evolutionary importance of the mechanism by these regulators operate. Indeed, the NodD amino acid sequence showed extensive conservation that ranged from > 90% sequence similarity among NodD proteins from organisms of the same genus to < 50% for NodD sequences from distantly related organisms (Göttfert *et al.*, 1992). Although the global sequence similarity for NodD can vary considerably among different organisms, a general feature is the high-to-low conservation in the amino acid sequence within the protein from the amino-terminus to the carboxy-terminus of the polypeptide. This structural pattern occurs in many rhizobia species and is consistent with the function attributed to each domain of the protein (Burn *et al.*, 1987, 1989; Horvath *et al.*, 1987).

The amino-terminal region, the most conserved part of the protein, anchors the HTH DNA-binding motif responsible for recognition of the *nod* box *cis*-elements in bacterial DNA. Despite this higher conservation in sequence similarity, subtle amino acid substitutions nevertheless occur among *B. elkanii* genomes and may ultimately modify the affinity of this motif for the DNA, thereby altering the pattern of binding and consequent activity of these regulators. On the other hand, the NodD carboxy-terminus region is believed to be primarily involved in protein oligomerization, an important aspect since it is generally accepted that NodD_1_ is active in a tetrameric form that arises from the cognate homodimers (Peck *et al.*, 2013). Multiple weak interactions occur among non-specific amino acids in this region of NodD proteins and may contribute to oligomerization (Ezezika *et al.*, 2007; Peck *et al.*, 2013). As observed in other rhizobia, the NodD carboxy-terminus region was clearly less conserved in the *B. elkanii* genomes. Even though this lower sequence similarity did not markedly affect protein structure, it may play a role in oligomerization.

**Figure 5:**
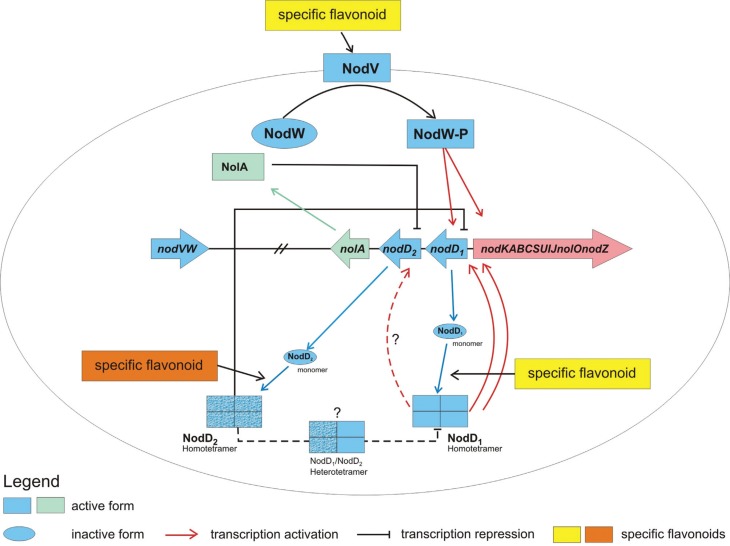
A model illustrating the modulation of *nod* gene expression proposed for *B. elkanii*. Expression of the *nod* operon containing the structural *nod* genes is regulated by regulatory *nod* genes (*nod* regulon) in the presence of the respective flavonoid inducer. Transcriptional activation of the *nod* operon is mediated by NodD_1_ and NodVW, resulting in the biosynthesis of lipochitooligosaccharides. Negative regulation of the *nod* operon is due to the action of NodD_2_ and indirectly by NolA. Based on Loh and Stacey (2003).

Based on the information available for the *B. diazoefficiens* USDA 110 reference genome, the similarities between *B. diazoefficiens* and *B. elkanii*, and the results obtained from this comparative genomic analysis, an extension in the model of *nod* gene regulation proposed for *B. diazoefficiens* can be applied to *B. elkanii* with some new added features (Figure 5). Besides the activity of all known elements in the core mechanism of *nod* operon regulation by the already discussed *nod* regulon, the presence of a partial *nod* box sequence upstream to *nodD*_*2*_ suggests a putative NodD_1_ binding site in this region, with a possible regulatory effect on *nodD*_*2*_ as a transcriptional activation. Such a mechanism may possibly function under higher concentrations of NodD_1_ as a consequence of the autoregulation of *nodD*_*1*_ expression by its respective product. Under these conditions, a surplus of NodD_1_ may be “trapped” by this partial *nod* box sequence; NodD_1_ may show lower affinity for the regulator protein relative to the conserved consensus *nod* box sequence and ultimately activate transcription of the repressor protein NodD_2_ to balance expression of the *nod* operon.

The prediction of highly conserved structures between NodD_1_ and NodD_2_ and their structural alignment suggested the possible formation of NodD_1_-NodD_2_ heterodimers since their protein structures are very similar, but no specific amino acid residues responsible for oligomerization have yet been identified. If NodD_1_-NodD_2_ heterodimers are formed in *Bradyrhizobium*, they may well affect the NodD_1_-driven regulation in this symbiont, in addition to interfering with *nod* operon gene expression. Although the data are suggestive of these events, there is still no conclusive evidence for this hypothesis and experimental confirmation of such mechanisms is required to prove the validity of this model.

Our knowledge of the genetic control of nodulation and our understanding of the mechanisms involved in the early events of a new symbiotic relationship have undoubtedly expanded considerably in the last two decades. Although a general framework for nodulation and symbiosis has not yet been developed for all diazotrophic microorganisms, given their genetic and phenotypic diversity, comparative studies may nevertheless help to clarify these processes and provide some useful preliminary evidence for future investigations. As microbial genome sequencing projects are quickly delivering an ever-increasing amount of data, comparative analyses of *cis*-elements and their cognate *trans*-acting factors could provide new insights into the activation and/or repression of *nod* gene expression.

This work has identified several similarities between *B. elkanii* genomes and the closely related *B. diazoefficiens*. Based on these similarities, it was possible to identify and correlate functional mechanisms and key elements that play an essential role in regulating *nod* gene expression. In addition, new genomic features that had not been clearly explored before, some of which were unique to certain *B. elkanii* genomes, have raised new questions for future research.

## Supplementary material

The following online material is available for this article:

Table S1 - *Bradyrhizobium* *nod* operon/regulon gene inventory.

Figure S1 - Alignment of the ~250-bp region upstream from *nolA* and *nodVW* ORFs.

Figure S2 - Evolutionary relationship of the *nod* regulatory proteins NodD_1_ and NodD_2._

Figure S3 - Global similarity of NodD regulator proteins in *B. elkanii* strains.

Figure S4 - Conservation plot from the multiple sequence alignment of NodD regulator proteins.

Figure S5 - NodD_1_ global protein structure alignment for *B. elkanii* strains against *B.* *diazoefficiens* USDA 110 using the Combinatorial Extension (CE) algorithm.

Figure S6 - NodD_2_ global protein structure alignment for *B. elkanii* strains against *B.* *diazoefficiens* USDA 110 using the Combinatorial Extension (CE) algorithm.

Figure S7 - NodD_1_-NodD_2_ global protein structure alignment within each *Bradyrhizobium* genome using the Combinatorial Extension (CE) algorithm.
