# Supplementary material for: Bradyrhizobium elkanii nod regulon: insights through genomic analysis
Source: Genet Mol Biol. 2017 Jul 31;40(3):703–16. doi: 10.1590/1678-4685-GMB-2016-0228 (PMC5596368; doi:10.1590/1678-4685-GMB-2016-0228)
Supplement: Supplementary file 1 [file 1415-4757-gmb-1678-4685-GMB-2016-0228-Suppl01.pdf]

Genetics and Molecular Biology, 40, 3 (2017)

Supplementary material to "Bradyrhizobium elkanii nod regulon: insights through genomic analysis"

Table S1. *Bradyrhizobium nod* operon/regulon gene inventory.

Organism	Gene			
	ID	Coordinates	nt	Aa
Bradyrhizobium diazoefficiens USDA 110	*nod*W*	BA000040.2_1878986_1879579	594	197
	*nod*V*	BA000040.2_1882335_1879666	2670	889
	*nol*Z	BA000040.2_2175594_2175821	228	75
	*nol*Y	BA000040.2_2175928_2176656	729	242
	*nol*A*	BA000040.2_2179134_2179706	573	190
	*nod*D2*	BA000040.2_2180766_2181758	993	330
	*nod*D1*	BA000040.2_2182392_2183336	945	314
	*nod*Y	BA000040.2_2183450_2184088	639	212
	*nod*K	-	-	-
	*nod*A	BA000040.2_2184130_2184762	633	210
	*nod*B	BA000040.2_2184963_2185416	453	151
	*nod*C	BA000040.2_2185433_2186890	1458	485
	*nod*S	BA000040.2_2186919_2187446	528	175
	*nod*U	BA000040.2_2187460_2189169	1710	569
	*nod*I	BA000040.2_2189186_2190091	906	301
	*nod*J	BA000040.2_2190095_2190883	789	262
	*nol*M	BA000040.2_2190916_2191089	174	57
	*nol*N	BA000040.2_2191127_2191522	396	131
	*nol*O	BA000040.2_2191558_2193165	1647	548
	*nod*Z	BA000040.2_2193331_2194443	1113	370
Bradyrhizobium elkanii SEMIA 587	*nod*W*	AJJK01000027.1_118833_119536	704	233
	*nod*V*	AJJK01000029.1_119623_122295	2673	890
	*nol*Z	-	-	-
	*nol*Y	AJJK01000096.1_326076_326309	234	77
	*nol*A*	AJJK01000095.1_321036_321522	487	162
	*nod*D2*	AJJK01002387.1_8523194_8524189	996	331
	*nod*D1*	AJJK01002387.1_8524830_8525774	945	314
	*nod*Y	-	-	-
	*nod*K	AJJK01002387.1_8526308_8526585	278	91
	*nod*A	AJJK01002387.1_8526625_8527257	663	210
	*nod*B	AJJK01002387.1_8527458_8527913	456	151
	*nod*C	AJJK01002387.1_8527930_8529303	1374	457
	*nod*S	AJJK01002387.1_8529419_8529946	528	175
	*nod*U	AJJK01000365.1_1215479_1216615	1137	378
	*nod*I	AJJK01000616.1_2107854_2108629	776	257
	*nod*J	AJJK01000616.1_2106915_2106706	792	263
	*nol*M	-	-	-
	*nol*N	-	-	-
	*nol*O	AJJK01000616.1_2104611_2106371	1761	586
	*nod*Z	AJJK01000616.1_2103328_2104320	993	330
Bradyrhizobium elkanii CCBAU 05737	*nod*W*	AJPV01000599.1_9181873_9182704	832	277
	*nod*V*	AJPV01000599.1_9178877_9181549	2673	890
	*nol*Z	-	-	-
	*nol*Y	AJPV01000548.1_8647548_8647781	234	77
	*nol*A*	AJPV01000548.1_8642134_8642706	573	190
	*nod*D2*	AJPV01000548.1_8640072_8641067	996	331
	*nod*D1*	AJPV01000548.1_8638487_8639431	945	314
	*nod*Y	-	-	-
	*nod*K	AJPV01000548.1_8637677_8637953	277	91
	*nod*A	AJPV01000548.1_8637004_8637636	633	210

Organism	Gene			
	ID	Coordinates	nt	Aa
	*nod*B	AJPV01000548.1_8636348_8636803	456	151
	*nod*C	AJPV01000548.1_8634958_8636331	1374	457
	*nod*S	AJPV01000548.1_8634315_8634842	528	175
	*nod*U	AJPV01000548.1_8632591_8633781	1191	396
	*nod*I	AJPV01000548.1_8631669_8632589	921	306
	*nod*J	AJPV01000548.1_8630874_8631665	792	263
	*nol*M	-	-	-
	*nol*N	-	-	-
	*nol*O	AJPV01000548.1_8628570_8630597	2028	675
	*nod*Z	AJPV01000548.1_8627315_8628279	965	321
Bradyrhizobium elkanii CCBAU 43297	*nod*W*	AJPW01000165.1_2853646_2854325	678	225
	*nod*V*	AJPW01000165.1_2850986_2853649	2664	887
	*nol*Z	-	-	-
	*nol*Y	AJPW01000249.1_3991990_3992223	234	77
	*nol*A*	AJPW01000249.1_3997065_3997637	573	190
	*nod*D2*	AJPW01000249.1_3998704_3999699	996	331
	*nod*D1*	AJPW01000249.1_4000340_4001284	945	314
	*nod*Y	-	-	-
	*nod*K	AJPW01000249.1_4001692_4002094	403	133
	*nod*A	AJPW01000249.1_4002135_4002767	633	210
	*nod*B	AJPW01000249.1_4002968_4003423	456	151
	*nod*C	AJPW01000249.1_4003440_4004813	1374	457
	*nod*S	AJPW01000249.1_4004929_4005456	528	175
	*nod*U	AJPW01000249.1_4005471_4007180	1710	569
	*nod*I	AJPW01000249.1_4007182_4008102	921	306
	*nod*J	AJPW01000249.1_4008106_4008897	792	263
	*nol*M	-	-	-
	*nol*N	-	-	-
	*nol*O	AJPW01000249.1_4009174_4011201	2028	675
	*nod*Z	AJPW01000249.1_4011492_4012485	994	330
Bradyrhizobium elkanii USDA 94	*nod*W*	JAFC01000092.1_6221570_6222249	678	225
	*nod*V*	JAFC01000092.1_6218910_6221573	2464	887
	*nol*Z	-	-	-
	*nol*Y	-	-	-
	*nol*A*	JAFC01000072.1_8259046_8259618	573	190
	*nod*D2*	JAFC01000072.1_8260685_8261680	996	331
	*nod*D1*	JAFC01000072.1_8262321_8263265	945	314
	*nod*Y	-	-	-
	*nod*K	JAFC01000072.1_8263673_8264076	403	133
	*nod*A	JAFC01000072.1_8264116_8264748	633	210
	*nod*B	JAFC01000072.1_8264949_8265404	456	151
	*nod*C	JAFC01000072.1_8265421_8266794	1374	457
	*nod*S	JAFC01000072.1_8266910_8267437	528	175
	*nod*U	JAFC01000072.1_8267971_8269161	1191	396
	*nod*I	JAFC01000072.1_8269163_8270083	921	306
	*nod*J	JAFC01000072.1_8270087_8270878	792	263
	*nol*M	-	-	-
	*nol*N	-	-	-
	*nol*O	JAFC01000072.1_8271155_8273182	2028	675
	*nod*Z	JAFC01000072.1_8273471_8274465	995	331
Bradyrhizobium elkanii USDA 3254	*nod*W*	AXAH01000065.1_7903638_7903988	351	116
	*nod*V*	AXAH01000065.1_7900642_7903314	2673	890
	*nol*Z	-	-	-
	*nol*Y	-	-	-
	*nol*A*	AXAH01000041.1_6058297_6058869	573	190
	*nod*D2*	AXAH01000041.1_6056239_6057228	990	329

Organism	Gene			
	ID	Coordinates	nt	Aa
	*nod*D1*	AXAH01000041.1_6054783_6055619	837	278
	*nod*Y	-	-	-
	nodK	AXAH01000041.1_6054210_6054472	263	86
	*nod*A	AXAH01000041.1_6053183_6053815	633	210
	*nod*B	AXAH01000041.1_6052527_6052982	456	151
	*nod*C	AXAH01000041.1_6051120_6052509	1386	461
	*nod*S	AXAH01000041.1_6050494_6051021	528	175
	*nod*U	AXAH01000041.1_6048771_6050480	1710	569
	*nod*I	AXAH01000041.1_6047849_6048769	921	306
	*nod*J	AXAH01000041.1_6047054_6047845	792	263
	*nol*M	-	-	-
	*nol*N	-	-	-
				675
	*nol*O	AXAH01000041.1_6044746_6046773	2028	
	*nod*Z	AXAH01000041.1_6043471_6044465	995	331
	*nod*W*	AXAW01000069.1_7664909_7665259	351	116
	*nod*V*	AXAW01000069.1_7661913_7664585	2673	890
	*nol*Z	-	-	-
	*nol*Y	-	-	-
	*nol*A*	AXAW01000040.1_5585979_5586551	573	190
	*nod*D2*	AXAW01000040.1_5587618_5588609	992	329
	*nod*D1*	AXAW01000040.1_5589229_5590065	837	278
	*nod*Y	-	-	-
	*nod*K	AXAW01000040.1_5590375_5590638	264	88
Bradyrhizobium elkanii USDA 3259	*nod*A	AXAW01000040.1_5591033_5591665	633	210
	*nod*B	AXAW01000040.1_5591866_5592321	456	151
	*nod*C	AXAW01000040.1_5592339_5593728	1386	461
	*nod*S	AXAW01000040.1_5593827_5594354	528	175
	*nod*U	AXAW01000040.1_5594368_5596077	1710	569
	*nod*I	AXAW01000040.1_5596079_5596999	921	306
	*nod*J	AXAW01000040.1_5597003_5597794	792	263
	*nol*M	-	-	-
	*nol*N	-	-	-
	*nol*O	AXAW01000040.1_5598075_5600102	2028	675
	*nod*Z	AXAW01000040.1_5600383_5601377	995	331

*regulatory *nod* genes *nodD1* and *nodD2*. The core regulatory genes in the *nod* regulon are highlighted in grey.
