# Supplementary material for: Bradyrhizobium elkanii nod regulon: insights through genomic analysis
Source: Genet Mol Biol. 2017 Jul 31;40(3):703–16. doi: 10.1590/1678-4685-GMB-2016-0228 (PMC5596368; doi:10.1590/1678-4685-GMB-2016-0228)
Supplement: Supplementary file 2 [file 1415-4757-gmb-1678-4685-GMB-2016-0228-Suppl02.pdf]

Genetics and Molecular Biology, 40, 3 (2017)

Supplementary material to "Bradyrhizobium elkanii nod regulon: insights through genomic analysis"

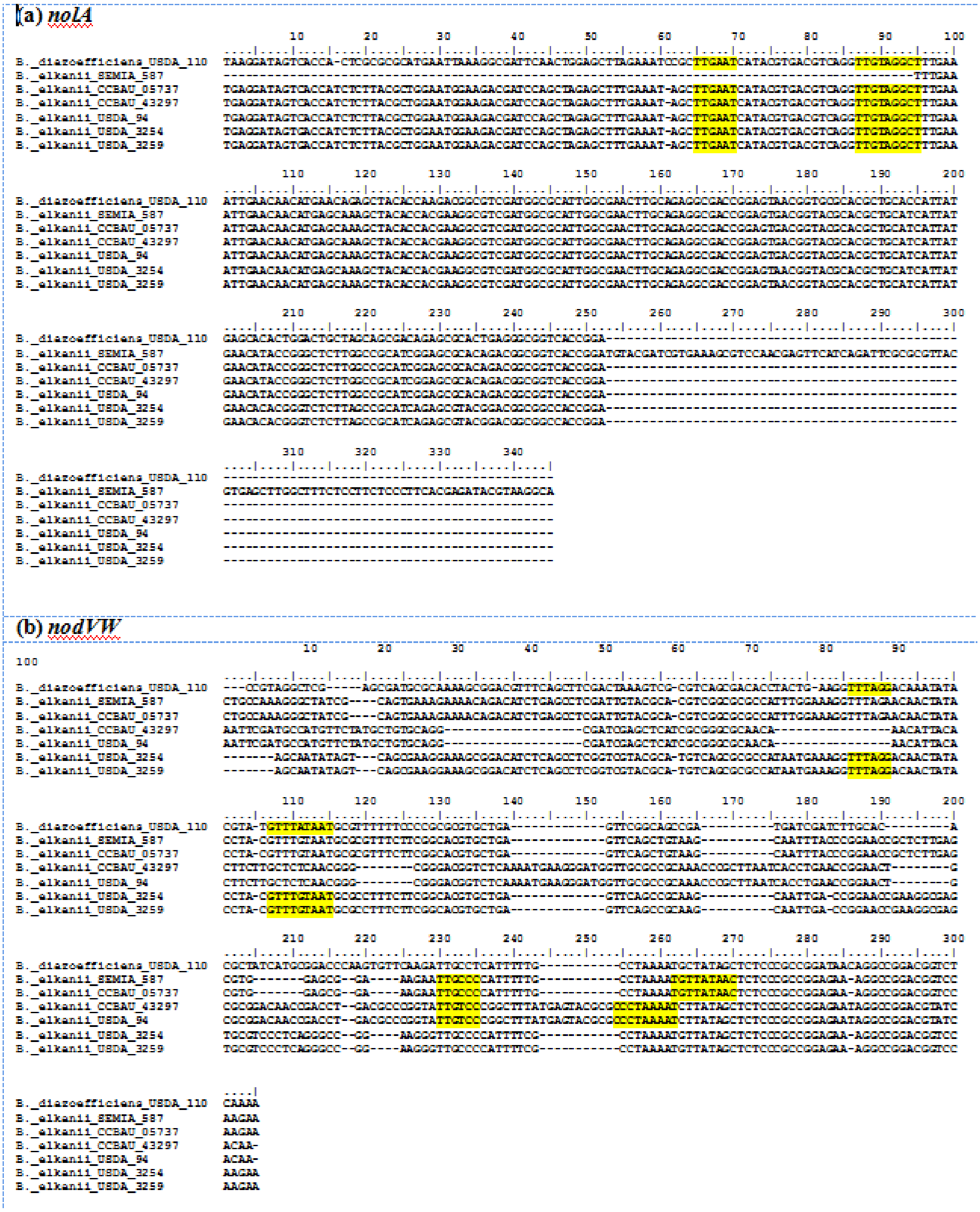

Figure S1 - Alignment of the ~250-bp region upstream from the *nolA* (a) and *nodVW* (b) ORFs in *Bradyrhizobium* genomes. Sequences highlighted in yellow show a -10/-35 σ^{70} potential promoter. No putative *nod* box motifs (ATC-N_9-GAT) were found in these promoter regions. Sequences read 5' to 3' from right to left, with the first nucleotide in the sequence representing that immediately prior to ATG from the CDS start site.
