# Supplementary material for: Bradyrhizobium elkanii nod regulon: insights through genomic analysis
Source: Genet Mol Biol. 2017 Jul 31;40(3):703–16. doi: 10.1590/1678-4685-GMB-2016-0228 (PMC5596368; doi:10.1590/1678-4685-GMB-2016-0228)
Supplement: Supplementary file 3 [file 1415-4757-gmb-1678-4685-GMB-2016-0228-Suppl03.pdf]

Genetics and Molecular Biology, 40, 3 (2017)

Supplementary material to "Bradyrhizobium elkanii nod regulon: insights through genomic analysis"

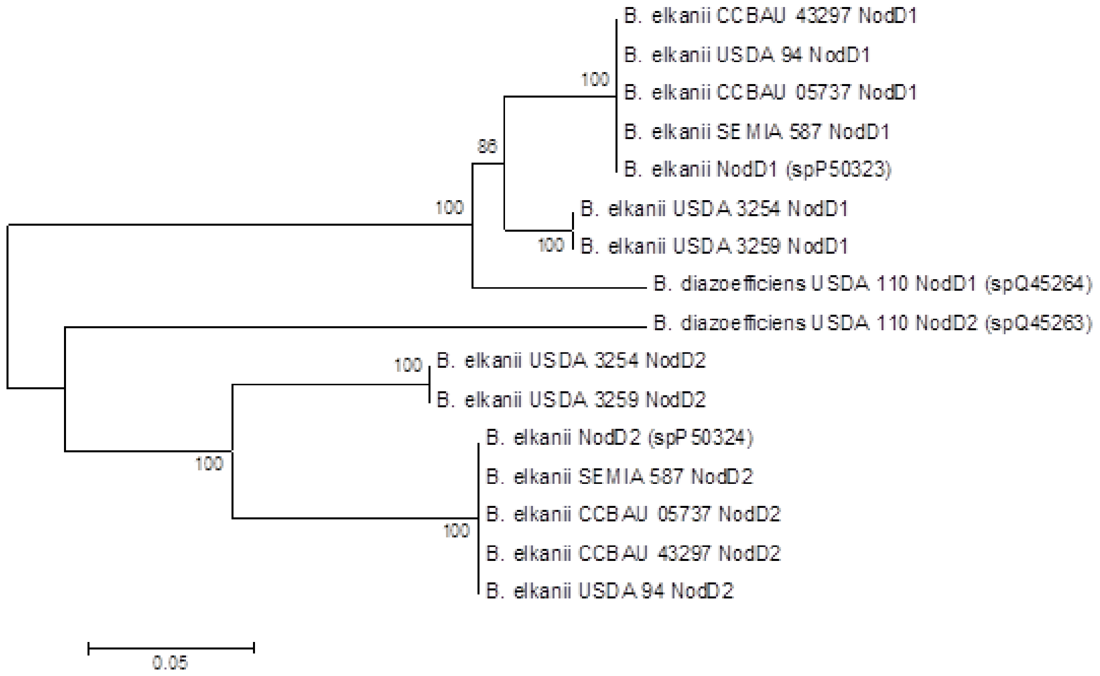

Figure S2 - Evolutionary relationship of the *nod* regulatory proteins $NodD_1$ and $NodD_2$ from various *B. elkanii* strains. The curated reference proteins $NodD_1$ (spP50323) and $NodD_2$ (spP50324) from *B. elkanii* have been included. The evolutionary history was inferred using the Neighbor-Joining method. The optimal tree with the sum of branch length = 0.63145170 is shown, with the percentage of replicate trees in which the associated taxa clustered together in the bootstrap test (1,000 replicates) shown next to the branches. The tree is drawn to scale, with branch lengths in the same units as those of the evolutionary distances used to infer the phylogenetic tree. The distances were computed using the p-distance method and were expressed as the number of amino acid differences per site. All ambiguous positions were removed for each sequence pair and there were 331 positions in the final dataset.
