# Supplementary material for: Bradyrhizobium elkanii nod regulon: insights through genomic analysis
Source: Genet Mol Biol. 2017 Jul 31;40(3):703–16. doi: 10.1590/1678-4685-GMB-2016-0228 (PMC5596368; doi:10.1590/1678-4685-GMB-2016-0228)
Supplement: Supplementary file 4 [file 1415-4757-gmb-1678-4685-GMB-2016-0228-Suppl04.pdf]

Genetics and Molecular Biology, 40, 3 (2017)

# Supplementary material to "Bradyrhizobium elkanii nod regulon: insights through genomic analysis"

**(a) $NodD_1$**

| | *B. diazoefficiens* USDA 110 | *B. elkanii* SEMIA 587 | *B. elkanii* CCBAU 05737 | *B. elkanii* CCBAU 43297 | *B. elkanii* USDA 94 | *B. elkanii* USDA 3254 | *B. elkanii* USDA 3259 |
|---|---|---|---|---|---|---|---|
| *B. diazoefficiens* USDA 110 | 1 | | | | | | |
| *B. elkanii* SEMIA 587 | 0.93 | 1 | | | | | |
| *B. elkanii* CCBAU 05737 | 0.93 | 1 | 1 | | | | |
| *B. elkanii* CCBAU 43297 | 0.93 | 1 | 1 | 1 | | | |
| *B. elkanii* USDA 94 | 0.93 | 1 | 1 | 1 | 1 | | |
| *B. elkanii* USDA 3254 | 0.94 | 0.93 | 0.93 | 0.93 | 0.93 | 1 | |
| *B. elkanii* USDA 3259 | 0.94 | 0.93 | 0.93 | 0.93 | 0.93 | 1 | 1 |

**(b) $NodD_2$**

| | *B. diazoefficiens* USDA 110 | *B. elkanii* SEMIA 587 | *B. elkanii* CCBAU 05737 | *B. elkanii* CCBAU 43297 | *B. elkanii* USDA 94 | *B. elkanii* USDA 3254 | *B. elkanii* USDA 3259 |
|---|---|---|---|---|---|---|---|
| *B. diazoefficiens* USDA 110 | 1 | | | | | | |
| *B. elkanii* SEMIA 587 | 0.74 | 1 | | | | | |
| *B. elkanii* CCBAU 05737 | 0.74 | 1 | 1 | | | | |
| *B. elkanii* CCBAU 43297 | 0.74 | 1 | 1 | 1 | | | |
| *B. elkanii* USDA 94 | 0.74 | 1 | 1 | 1 | 1 | | |
| *B. elkanii* USDA 3254 | 0.73 | 0.88 | 0.88 | 0.88 | 0.88 | 1 | |
| *B. elkanii* USDA 3259 | 0.73 | 0.88 | 0.88 | 0.88 | 0.88 | 1 | 1 |

**Figure S3 -** Global similarity of NodD regulator proteins in *B. elkanii* strains assessed using a Percent Accepted Mutations (PAM250) matrix. Boxes shaded from dark to light gray represent higher to lower sequence similarities, respectively. The analysis was done for $NodD_1$ (a) and $NodD_2$ (b) protein sequences.
