# Supplementary material for: Bradyrhizobium elkanii nod regulon: insights through genomic analysis
Source: Genet Mol Biol. 2017 Jul 31;40(3):703–16. doi: 10.1590/1678-4685-GMB-2016-0228 (PMC5596368; doi:10.1590/1678-4685-GMB-2016-0228)
Supplement: Supplementary file 5 [file 1415-4757-gmb-1678-4685-GMB-2016-0228-Suppl05.pdf]

Supplementary material to "Bradyrhizobium elkanii nod regulon: insights through genomic analysis"

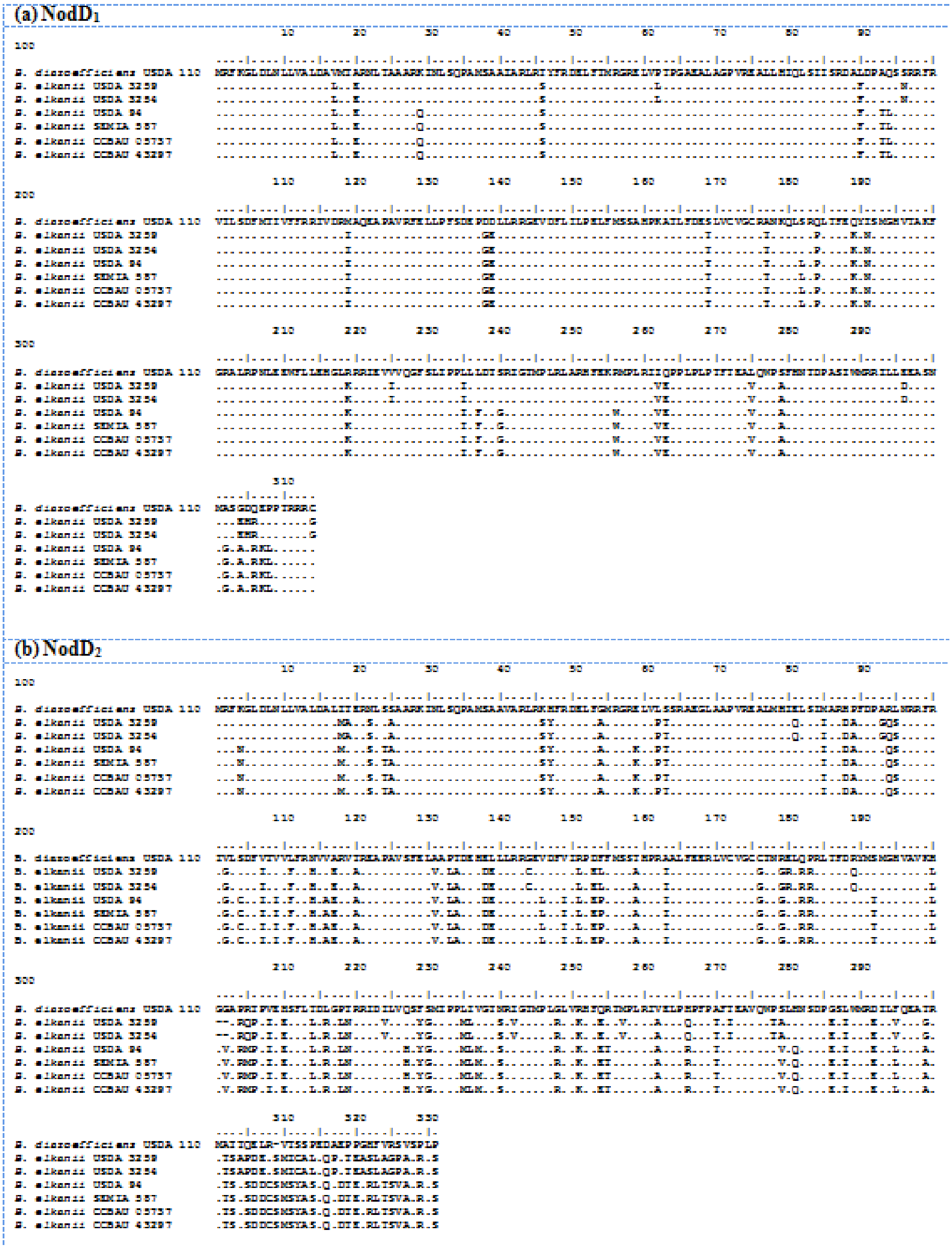

Figure S4 - Conservation plot from the multiple sequence alignment (MSA) of NodD regulator proteins from the genomes of different *B. elkanii* strains compared to the *B. diazoefficiens* USDA 110 reference genome. Global protein alignment was done using the **Mu**ltiple **S**equence **C**omparison by **L**og-**E**xpectation (MUSCLE) algorithm for $NodD_1$ (a) and $NodD_2$ (b) protein sequences.
