# Supplementary material for: Bradyrhizobium elkanii nod regulon: insights through genomic analysis
Source: Genet Mol Biol. 2017 Jul 31;40(3):703–16. doi: 10.1590/1678-4685-GMB-2016-0228 (PMC5596368; doi:10.1590/1678-4685-GMB-2016-0228)
Supplement: Supplementary file 6 [file 1415-4757-gmb-1678-4685-GMB-2016-0228-Suppl06.pdf]

Genetics and Molecular Biology, 40, 3 (2017)

Supplementary material to "Bradyrhizobium elkanii nod regulon: insights through genomic analysis"

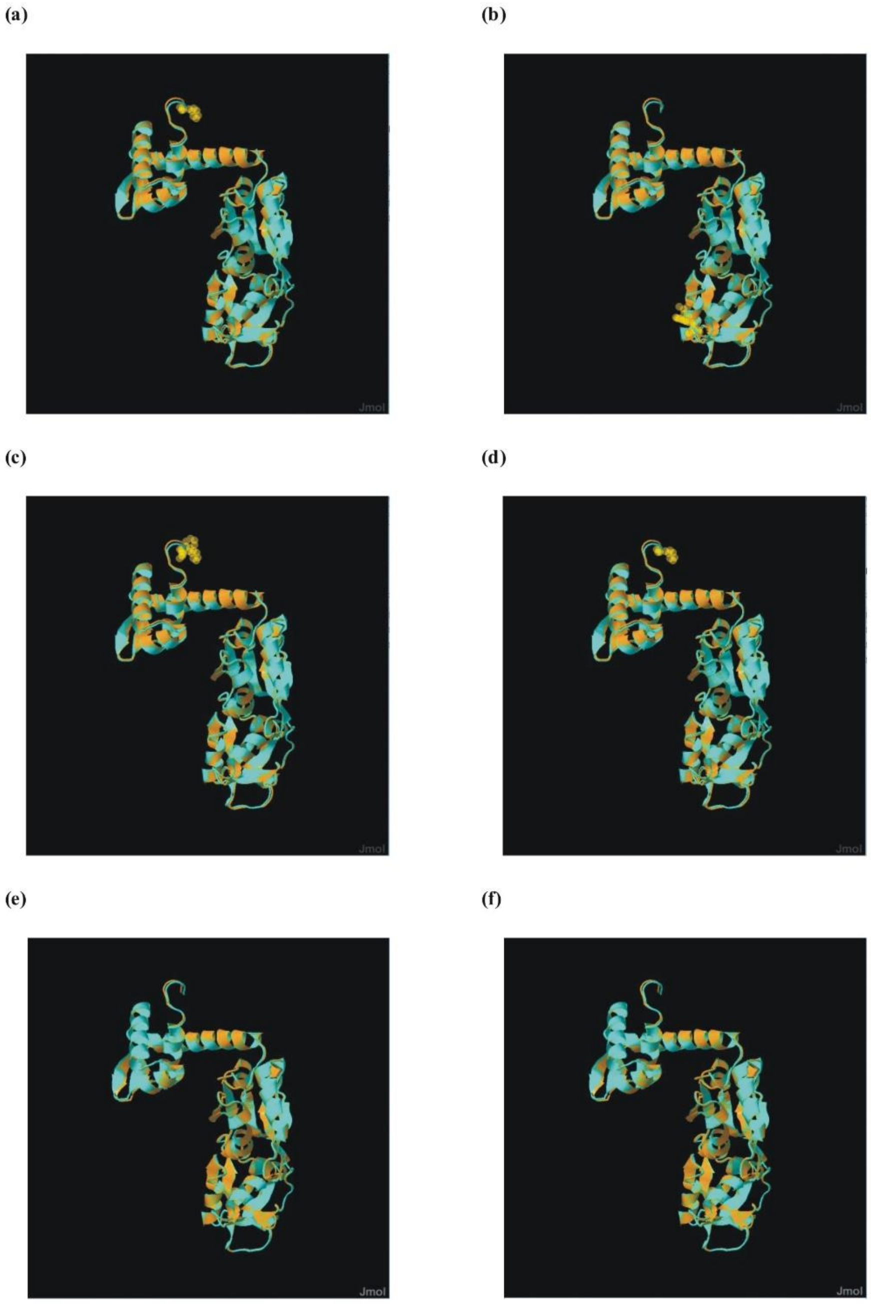

Figure S5 - $NodD_1$ global protein structure alignment for *B. elkanii* strains against *B. diazoefficiens* USDA 110 using the Combinatorial Extension (CE) algorithm. Orange residues represent $NodD_1$ from *B. diazoefficiens* USDA 110 superimposed on light-blue residues representing $NodD_1$ from *B. elkanii* SEMIA 587 (a), *B. elkanii* CCBAU 05737 (b), *B. elkanii* CCBAU 43297 (c), *B. elkanii* USDA 94 (d), *B. elkanii* USDA 3254 (e) and *B. elkanii* USDA 3259 (f).
